# A multi-robot deep Q-learning framework for priority-based sanitization of railway stations

**DOI:** 10.1007/s10489-023-04529-0

**Published:** 2023-04-18

**Authors:** Riccardo Caccavale, Mirko Ermini, Eugenio Fedeli, Alberto Finzi, Vincenzo Lippiello, Fabrizio Tavano

**Affiliations:** 1 Department DIETI, Università degli Study di Napoli “Federico II”, via Claudio 21, Naples, 80125 Italy; 2 Department Research and Development, Rete Ferroviaria Italiana, Via Curzio Malaparte 8, Firenze Osmannoro, 50145 Italy; 3 Department Research and Development, Rete Ferroviaria Italiana, Piazza della Croce Rossa 1, Roma, 00161 Italy; 4 Department Research and Development, Rete Ferroviaria Italiana, Via del Portonaccio 175, Roma, 00159 Italy

**Keywords:** Deep Q-network, Convolutional neural network, Heatmap, Decentralized, Multi-agent, Sanitization

## Abstract

Sanitizing railway stations is a relevant issue, primarily due to the recent evolution of the Covid-19 pandemic. In this work, we propose a multi-robot approach to sanitize railway stations based on a distributed Deep Q-Learning technique. The proposed framework relies on anonymous data from existing WiFi networks to dynamically estimate crowded areas within the station and to develop a heatmap of prioritized areas to be sanitized. Such heatmap is then provided to a team of cleaning robots - each endowed with a robot-specific convolutional neural network - that learn how to effectively cooperate and sanitize the station’s areas according to the associated priorities. The proposed approach is evaluated in a realistic simulation scenario provided by the Italian largest railways station: Roma Termini. In this setting, we consider different case studies to assess how the approach scales with the number of robots and how the trained system performs with a real dataset retrieved from a one-day data recording of the station’s WiFi network.

## Introduction

Modern societies are increasingly open and connected, the demand for transport is continuously increasing [1] and the high volume and frequency of passengers moving between cities is an evident cause for rapid spread of diseases across countries [2]. In this regard, the role of railway stations is crucial. Stations are not only nodes of the railway network where passengers are in transit, but also locations for shops, restaurants, places for recreation and aggregation [3, 4]. Passengers gathering in the halls and platforms of the stations, eating at restaurants, and boarding the trains facilitate the transmission of diseases [5]. For this reason, the pandemic caused by the SARS-CoV-2 has spawned a crisis that has affected the railway sector in a significant way [6], for example, by inducing people to prefer cars instead of trains [7]. It is then strategic for the infrastructure managers (such as the Italian Rete Ferroviaria Italiana) to deploy adequate and modern tools to prevent future contagion inside railway stations [8]. In this scenario, disinfectant robot technologies can support in fighting pandemics [9] by reducing the number of people involved in the cleaning process and by enhancing the sterilization performance. In this direction, we propose an approach to multi-robot sanitization suitable for teams of robots capable of cleaning large human-populated railway stations [10]. Specifically, we present a distributed Deep Q-Learning method to generate effective cleaning strategies that drive a set of sanitizing robots towards the most critical regions of the station. Such regions are prioritized by estimating the most populated areas of the station to be sanitized. For this purpose, we rely on the WiFi infrastructure, which is often available in public environments, to retrieve the position of the smartphones [11] to get an approximated assessment of the distribution and concentration of the crowd. Such estimated distribution of people in the areas of the railway station is then used to specify a heatmap of prioritized areas to be sanitized, which is dynamically updated and provided to each of the robots to enable them to continuously adapt their cooperative cleaning behaviors. It should be noted that cleaning railway stations in the presence of humans represents a very challenging real-world domain for multi-robot coordination, not only because of the large environment to be effectively covered by multiple agents, but also for the continuous variation of the people distribution that requires dynamic adaptation of the target areas to be reached. In this scenario, we propose to deploy a distributed Deep Q-Learning approach, where each robotic agent, endowed with a specific Deep Q-Network (DQN), learns how to adapt its cleaning behavior with respect to the shared estimated heatmap. For each robot, given the current heatmap and position, the generated strategy provides the next adjacent area to sanitize in order to maximize the overall team reward. Notice that in this paper we focus on the decision and coordination problem (i.e., areas to be cleaned by the robots), while we assume that each cleaning robot is endowed with a suitable navigation system (localization, obstacle avoidance, path and motion planning systems, etc.) to safely reach the areas established by the cleaning strategy. The peculiarity and originality of the approach relies on the exploitation of the shared dynamic heatmap in combination with a distributed deep learning algorithm, where each agent can independently learn how to effectively sanitize a large and populated environment in cooperation with other agents. While the distributed algorithm permits to scale with the number of robots, the shared heatmap accounts for dynamically updated priorities in large populated environments, which are rarely considered in multi-agent sanitizing frameworks. In our setting, such priorities are associated with the dynamics of contaminated areas, which is modeled by a Gaussian convolution law. We investigate the system at work in several realistic case studies defined in the context of the Roma Termini railway station. In this scenario, we evaluate the system performance exploiting both simulated and real data about the people distribution inside the station. The collected results show that the proposed approach is feasible in real-world scenarios, competitive with respect to alternative methods, and scalable with respect to the number of sanitizing agents and the crowding of the station. The main contributions of this work can be summarized as follows:

- We propose a scalable framework, where multiple mobile robots independently learn to cooperate for the execution of cleaning tasks in crowded indoor environments.
- The proposed method relies on the station’s WiFi infrastructure to monitor the distribution of people in the environment and estimate the diffusion of contaminants, thus allowing to dynamically prioritize the areas to be sanitized.
- We introduce and discuss several case studies in a real-world challenging environment provided by the Roma Termini railway station. In this scenario, we show how the proposed system responds to online priority changing and how it scales with respect to the number of robots and the crowding of the station.
- We discuss the framework at work with a real dataset in which the distribution of people is retrieved from a one-day data recording provided by the WiFi Network of Roma Termini.
- The performance of the proposed system is also compared with respect to standard coverage path planning techniques.

The rest of the paper is structured as follows. In Section 2, we discuss literature related with multi-robot cleaning/sanitizing and area covering strategies. In Section 3, we describe the architecture of the proposed framework, the overall learning process, and the dataset used for testing. In Section 4, we present the case studies and discuss the collected empirical results. Finally, Section 5 concludes the paper and outlines some future directions of research.

## Related works

Several approaches have been proposed in the literature to design multi-robot strategies for cleaning activities. Different frameworks rely on coverage path planning (CPP) methods to reach and sanitize a specific area. Random walk methods [12] have been investigated to randomly and rapidly explore free regions exploiting multiple robotic agents, but these techniques are usually not efficient since robot overlapping and path repetitions are frequent. Other methods are based on fixed-shaped paths (spirals, rectangles, etc.), where each robot is assigned to a specific area of the environment [13–20]. These approaches are effective in providing a continuous cleaning service that maximizes the coverage and minimizes the idleness of the agents, on the other hand, they are not adaptive and do not provide dynamic prioritization mechanisms with respect to changing environments. In contrast, we are interested in generating sanitization strategies that take into account the dynamic evolution of the cleaning priorities. In this regard, we estimate the spread of contaminants by monitoring the distribution of people in the station to guide the robots towards sanitizing the most contaminated areas. Related works on CPP consider priorities during patrolling or surveillance tasks. For instance, in [21], the authors focus on the task of patrolling a given environment exploiting multiple agents and ensuring that static prioritized locations are visited within a predefined time period. Similarly, in [22–24], a surveillance task is addressed as a periodical revisit problem of a discrete set of sites associated with static priorities or fixed visitation frequencies. These approaches often consider static priorities and graph-based representations of the environments with a limited number of nodes. In contrast, we propose an approach that permits to dynamically update the robots’ behavior with respect to the estimated people distribution in the environment and the associated risk of contaminant. Moreover, we are interested in providing an approach suitable for high resolution priorities in very large railway stations and scalable with respect to the number of robots in the team. In particular, Reinforcement Learning (RL) approaches have been deployed in the context of cleaning or disinfection of structured environments based on double DQN [13] or Actor-Critic Experience Replay (ACER) [17]. These approaches are more flexible than the ones based on CPP, but usually, only single-robot setups are considered. On the other hand, Multi Agent Reinforcement Learning (MARL) frameworks are often proposed to ensure flexibility and scalability in different applications like exploration [25], construction [26], or target-capturing [27], while priority-based cleaning issues are less explored. For instance, in [25], the authors proposed a cooperative multi-agent Deep Reinforcement Learning technique to solve multi-target capturing tasks, where a unique end-to-end network is deployed and trained over local robot-centered portions of the environment from each agent. Similarly, in [26], the authors presented a cooperative multi-robot exploration framework exploiting a two-phase multi-task Reinforcement Learning approach with cautiously-optimistic learners in combination with deep recurrent Q-networks for action-value approximation. Here, each robot is endowed with a specific neural network trained on local information from onboard sensors, supporting the modularity and the scalability of the framework. In [28], the authors developed a method to implement path planning and obstacle avoidance based on deep Q-learning with experience replay and a specific heuristic, which guides the robots to avoid the blind exploration of the action space during the training process. On the other hand, since the behavior and the distribution of people in a railway station are hardly predictable, the definition of such heuristic can be challenging. Similarly to our approach, a distributed framework is proposed in [27], where multiple agents learn a collaborative policy in a shared environment using the A3C training method for a construction task. Each agent perceives the other agents as moving features of the environment (hence no explicit communication is exploited). The effects of the actions are then detected by agents as disturbances to be compensated during the construction task, while the cooperative policy arises from the shared goal. Differently, in [29], the authors consider a decentralized MARL problem where the agents are connected via a time-varying and possibly sparse communication network. This method introduces a complex communication between robots, which have to merge local information from onboard sensors with the observations from the other agents of the team, and may lead to communication overhead [30]. In contrast, we are interested in investigating the case in which the information about the whole environment is shared between the robots through the heatmap exploiting the WiFi infrastructure available in the railway stations. Such heatmap is used to coordinate the agents hence no further explicit communication is needed. A similar approach is also described in [31], where the authors propose a multi-robot path planning algorithm using Deep Q-Learning combined with a Convolution Neural Network (CNN). In this case, a single image is used to represent the map, the obstacles, and the position of the agents in the environment, while the CNN is deployed to produce suitable paths for each robot in order to reach a single target location. Analogously to our work, the authors propose multi-agent Deep Q-learning, however they address a path planning problem with fixed targets, while we are interested in cleaning/sanitizing tasks with dynamically changing prioritized maps.

## The architecture

**Fig. 1:**
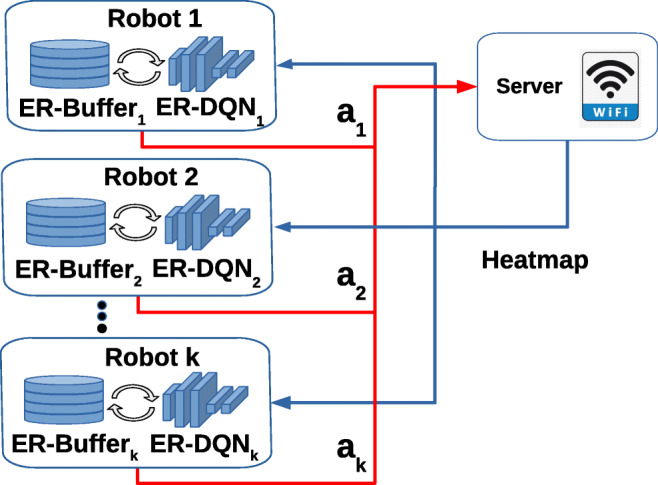
Graphical representation of the framework including multiple robotic agents (left) endowed with agent-specific experience replay buffers and neural networks, and a single server (right) exploiting WiFi data to generate a heatmap of priorities (red to yellow spots) shared by the robotic agents. Each robotic agent is equipped with a DQN that receives as input the shared heatmap along with the current robot position to learn the sanitizing policy (i.e, the next $a_{1},\dots ,a_{k}$ actions to execute). Actions are then communicated to the server, which updates the shared heatmap by resetting the cleaned cells

The multi-robot DQN approach proposed in this work is based on a decentralized client-server architecture where a team of *k* robots (clients) interacts with a central system (server) that maintains and updates a shared heatmap, whose hot spots are areas to be sanitized (see Fig. 1). In this setting, each robotic agent is equipped with a robot-specific DQN to learn how to adapt its cleaning behavior with respect to the shared heatmap. More specifically, for each robot, the associated DQN receives as input the current shared heatmap along with the robot position to generate a policy that provides the next adjacent area to sanitize. The shared heatmap maintained by the server can be described as follows. We assume the server endowed with a map of the environment defined by a 2-dimensional occupancy gridmap whose cells represent 1*m*^2^ areas of the station. Each cell of the gridmap is associated with a priority level, which depends on the presence of people on that cell, that indicates how risky the area is and how urgently the robots should sterilize it. Such priority levels on the gridmap are represented as a heatmap. Given such heatmap, the goal of the agents is to suitably navigate the gridmap by cleaning the traversed cells in order to reduce risky areas and the associated priorities. To tackle this problem we propose a distributed multi-robot Deep Q-Learning approach [31], where the involved robots are to independently learn coordinated and effective cleaning policies. Given a 2-dimensional occupancy gridmap *M* generated from the station’s planimetry, we denote by *X* the set of free-obstacle cells in *M* where a robot can be located. Each robot of the team can execute a set of actions *A*, where *a*_*i*_ ∈ *A* moves the robotic agent *i* from the current cell to an adjacent one (we assume 8 possible chess-king-like movements). The gridmap *M* is associated with a set of possible heatmaps *S*, each representing priority distributions on the gridmap cells. The goal of our learning problem is to generate, for each robot *i*, a suitable policy $\pi _{i} : S \times X \rightarrow A$ that maps agent positions *x*_*i*_ ∈ *X* and heatmaps *s* ∈ *S* into robot-specific actions *a*_*i*_ ∈ *A* driving the agent from the current cell to the next adjacent one to be sanitized.

A representation of the overall architecture is illustrated in Fig. 1. The framework is composed of a set of agents, representing mobile cleaning robots, each one communicating with the central server. The role of the server (server-side) is to merge the outcomes of the agents’ activities with (anonymized) data about people’s positions in order to produce the heatmap for the risky areas to be sterilized. The role of each agent (agent-side) is to receive heatmap and position from the server, update the robot policy exploiting the agent-specific DQN, and generate the next action to be executed.

### Heatmap

Heatmap-based models are quite common in literature to represent the spreading of contaminants in indoor environments [32, 33] as well as the distribution and the aggregation of people [34–39]. In this framework, we rely on a heatmap, whose hot/cold points represent high/low priority areas to be sanitized depending on the estimated distribution of people on the environment. The heatmap is then exploited by each sanitizing robot *i* in order to establish the next area to sterilize according to their specific policy *π*_*i*_. Specifically, each robotic agent *i* is associated with a DQN that receives as input a state-position couple (*s*,*x*_*i*_) ∈ *S* × *X*, which is represented as a 2-channel matrix *m* × *n* × 2, where *m* and *n* stand for the width and the height of the gridmap. The first channel *s* is a *m* × *n* matrix representing the heatmap (i.e., the cleaning priorities over the different grids). Each matrix element of the heatmap is a real number in the interval [0,1], where 1 stands for the maximum priority, while 0 means that no cleaning is needed. This matrix is displayed as a color-coded image (see heatmap in Fig. 1), where black pixels are 0 priority areas, while colors from red to yellow are for increasingly higher priorities. The second channel *x* is a binary *m* × *n* matrix representing the position and size of the current cleaning area occupied by the robot, which is 1 for the portions of the environment that are currently in the range of the robot cleaning effect, and 0 otherwise.

**Fig. 2:**
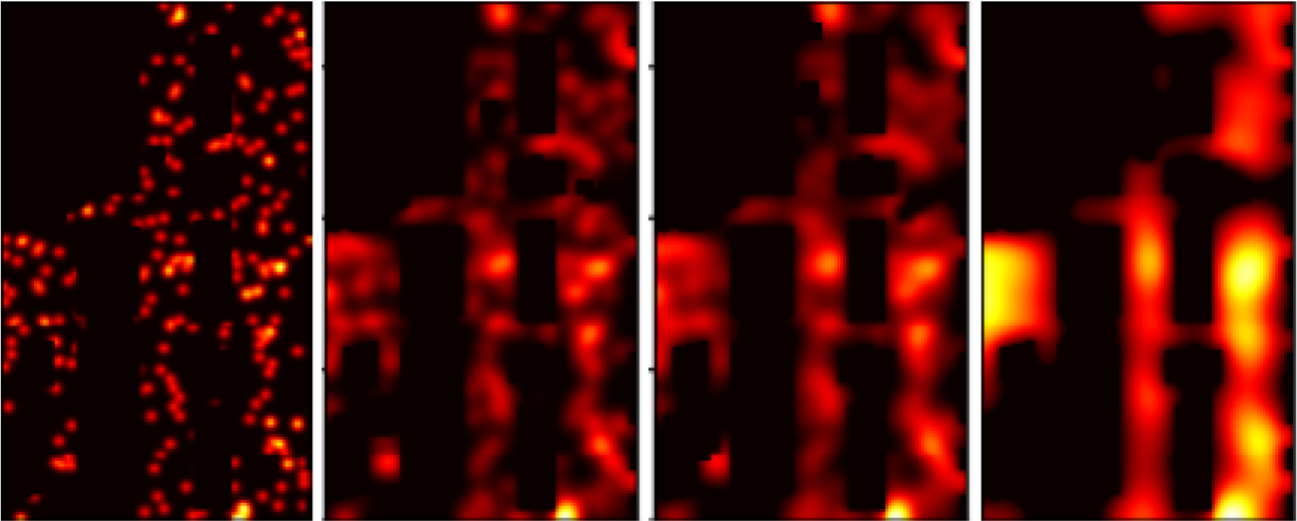
Evolution of the priority distribution starting from a random configuration of clusters. The pictures are collected every 10 steps from left (older) to the right (newer). Large black areas are static obstacles to be avoided/ignored by agents

In our framework, the update of priorities is performed by the server, which collects the outputs of the individual agents and integrates them taking into account the position of people and obstacles. As for the update of the heatmap (cleaning priority), it is computed from the position of group of people (clusters) by modeling possible spreading of viruses or bacteria using a Gaussian model of dispersion [40]. Specifically, we exploit the periodic convolution of a Gaussian filter $\mathcal {N}(\mu ,\sigma ^{2})$ every *ψ* steps, where *μ*, *σ*^2^ and *ψ* are suitable parameters that can be regulated depending on the meters/pixels ratio, the timestep, and the considered typology of spreading (in this work we assume a setting inspired by the aerial diffusion of the Covid-19 [41]). In our case, we set the *μ* and *σ* values according to the spreading parameters proposed in [42, 43]. Notice that since the heatmap update is computed from clusters of people by applying a simulated process, we do not need exact real-time people tracking in the environment, but only an approximated estimate of clusters’ positions and movements in railway station areas. An example of the evolution of a heatmap is proposed in Fig. 2. Here, starting from a set of static clusters, the probability distribution evolves through the iterative convolution of the Gaussian filter. The convolution process acts at every step by incrementally reducing the magnitude of the elements of the heatmap and distributing such priority over a larger area. This process is then exploited to simulate the effects of the attenuation such as the spreading of contaminants over time. In Fig. 2 there are also several fixed black areas that are associated with 0 priority, which correspond to the static obstacles retrieved from the map *M*. Such unreachable areas are assumed to be always clean, therefore unattractive and implicitly avoided by the robots. During the execution, when a robot *i* applies an action *a*_*i*_, it moves to a new cell sterilizing also all the cells that are in the cleaning range of the one reached. This behavior is also emulated by the server, which receives the executed actions from the agents and cleans the corresponding grids by resetting their associated priority to 0.

**Fig. 3:**
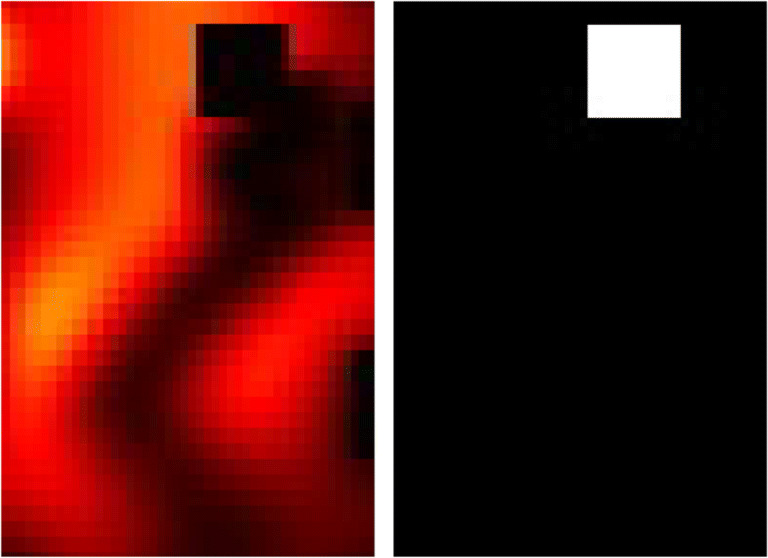
Zoomed cut of the 2-channels matrix representing the priority distribution (left) and position and size of the cleaning range of a single agent (right). The motion of the agent produces a low-priority wake in the traversed cells

In order to clarify this process, a portion of the two channels is illustrated in Fig. 3. In the first channel (left), it is possible to notice that as long as the agent moves through the environment it leaves a wake of cleaned space behind, that is proportional to the cleaning range in the second channel (right). This way, since the priority of already visited areas is 0, agents can indirectly observe their mutual behavior from the priority update, in so avoiding explicit communication.

### Multi-agent experience replay

**Fig. 4:**
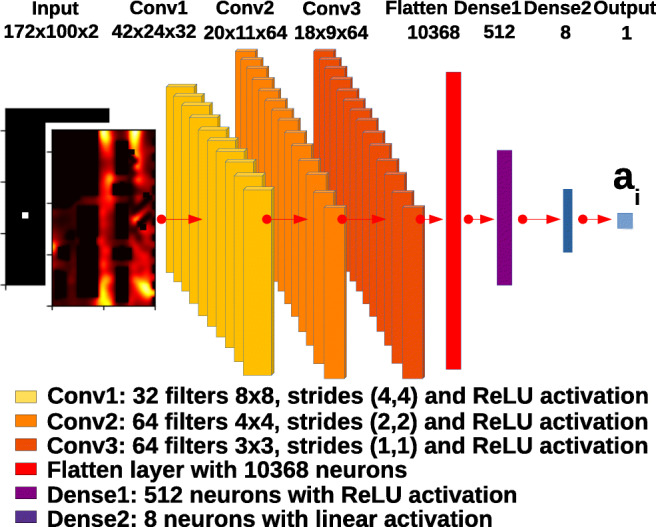
Structure of the convolutional neural network used by the robots. Dimensions are reported on top of each layer

In our framework, we propose a multi-agent variation of the experience replay method proposed in [31, 44]. In particular, our training scheme in this multi-agent setting follows a Distributed Training Decentralized Execution (DTDE) approach [45] where each robot updates its own individual policy independently from the others and without explicit information exchange between agents. Each of the *k* agents is then endowed with a specific replay buffer along with specific *target* and *main* DQNs, which are updated synchronously with respect to the position of the agent and the shared environment provided by the server (see Fig. 1). In particular, for the target and the main networks we deploy a convolutional neural network composed of the following layers: the first layer is a 2D convolutional layer with 32 filters 8 × 8, strides (4,4) and ReLU activation; the second is a 2D convolutional layer with 64 filters 4 × 4, strides (2,2) and ReLU activation; the third is a 2D convolutional layer with 64 filters 3 × 3, strides (1,1) and ReLU activation; the fourth is a Flatten layer; the fifth layer is a dense layer of 512 neurons still with ReLU activation; finally, the output layer is a dense layer composed of 8 neurons with linear activation. As for the update of the networks, we exploit the Adam optimizer with a learning rate *α* = 0.00025. A graphical representation of such networks is illustrated in Fig. 4. The 2-channel matrix is firstly processed by the 3 2D convolutional layers then the 3 dense layers are deployed to provide the 8 values of confidence for the 8 possible actions. The id the robot’s next action *a*_*i*_ is finally selected as the *argmax* of those 8 values.

### The learning process

As introduced in the previous Sections, the proposed learning procedure is composed of an *agent-side* component, where agent-specific training and execution processes take place, and a *server-side* component, where the overall team performance is evaluated and the state heatmap is updated.

In correspondence to these two sides, we define a local reward function *r*_*i*_ (agent-side), which is exploited by each agent to evaluate its performance during the cleaning activity, and a global reward function $r = {{\sum }_{i}^{k}} r_{i}$ (server-side) to summarize and evaluate the team performance. The local *reward* function *r*_*i*_ is designed to drive the agents toward prioritized areas of the environment (hot points), while avoiding obstacles and already visited areas (cold points). In this direction, we firstly introduce a cumulative priority function *cp*_*i*_ that summarizes the importance of a cleaned area:

1 $$ cp_{i} = \sum\limits_{(j,l)} s(j,l) x_{i}(j,l) $$

which is the sum of the element-wise priorities from matrix *s* in the area sterilized by the agent *i*. Specifically, for each grid of the map (*j*,*l*) the associated priority value *s*(*j*,*l*) is summed iff. it has been sanitized by the robot, i.e., when the grid is inside the cleaning range of the robot (*x*_*i*_(*j*,*l*) = 1).

The value in (1) is then exploited to define the reward *r*_*i*_ for the agent *i*:

2 $$ r_{i} = \left \{\begin{array}{ll} cp_{i} & \text{if $cp_{i} > 0$;} \\ penalty & \text{otherwise.} \end{array}\right. $$

Specifically, when an agent *i* sanitizes a prioritized area, the reward is equal to the cumulative value *cp*_*i*_; otherwise, if no priority is associated to the cleaned area (i.e., *cp*_*i*_ = 0) a negative reward *penalty* < 0 is earned (we empirically set *penalty* = − 2 for our case studies). This way, agents receive a reward that is proportional to the importance of the sanitized area, while routes toward zero-priority areas, such as obstacles or clean regions, are discouraged. Notice that in this framework, when the action of an agent leads to an obstacle (collision), no motion is performed. This behavior penalizes the agent (no further cleaning is performed), thus producing an indirect drive toward collision-free paths.

The learning procedure for a single robot is described in Algorithm 1. The Algorithm receives as input the id of the agent *i* and the occupancy gridmap of the environment *M* used to check actions’ feasibility. For each episode, each agent *i* selects an initial random position (lines 1-4), which is communicated to the server (line 5). At the beginning of each step, a new heatmap *s* is received from the server (line 7), which is used to select a new action *a*_*i*_ with an *𝜖*-probability of a random choice (line 8). The action is then emulated to observe a new agent-specific state $(s^{\prime }_{i}, x^{\prime }_{i})$ and a new reward *r*_*i*_ as specified by (2). The position $x^{\prime }_{i}$ is communicated to the server in order to be integrated into the next heatmap (line 10). The server checks for the task accomplishment (line 11), and the experience replay buffer is then suitably updated (line 12). Finally, if a terminal state is reached (i.e., the variable *done* is true) a new episode is started (lines 13-15).

**Algorithm 1:**
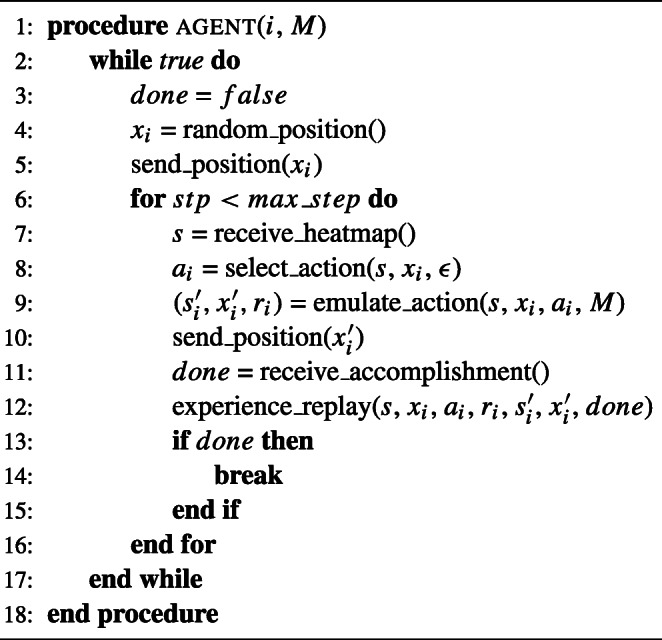
Agent-side learning algorithm.

The server-side learning process is described in Algorithm 2. In this case, when a new episode starts, the random positions of the agents are received (lines 1-4), and a new random heatmap *s* is produced (line 5). In particular, the random heatmap is created by generating random clusters of people in the free cells of the occupancy gridmap *M*. At the beginning of each step, a new heatmap *s* is sent to the agents (line 7). Each agent exploits the heatmap to select a new action according to its policy (see agent-side Algorithm 1, lines 7-10) sending back to the server the newly reached position. These positions are then received by the server (line 8) and used to update the heatmap by resetting (cleaning) the associated priorities (line 9). If the updated heatmap is sufficiently clean (we assume that the task is finalized when 98*%* of the map is clean) the task-accomplishment is communicated to the agents (lines 10-13) and a new episode can be started (lines 14-16). Otherwise, if the map is still not clean, the Gaussian spreading of contaminants is simulated (line 17).

**Algorithm 2:**
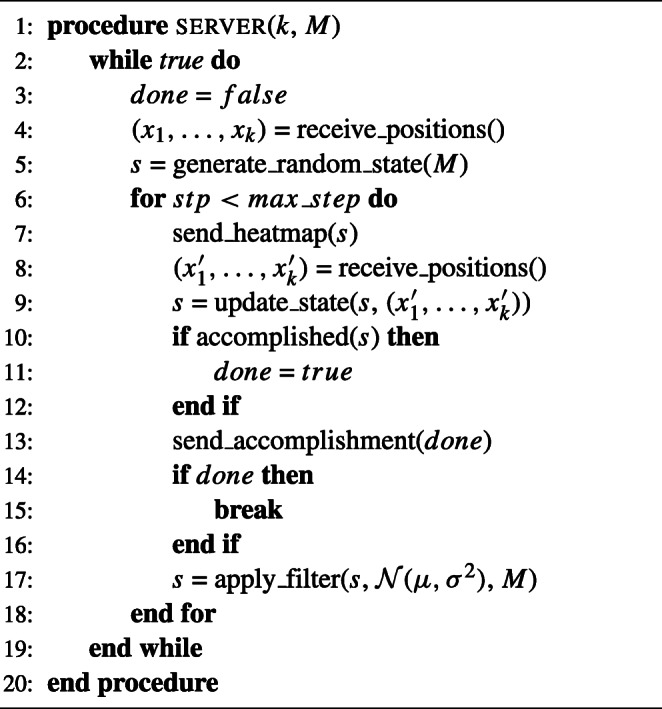
Server-side learning algorithm.

### The real-case dataset

In order to verify the performance of our approach, we collected a one-day real-data recording from the Meraki Cisco System WiFi Network of the Roma Termini station. The dataset contains information about the position and the distribution of the people in the station within a 24 hours interval starting from 22:00 on 12 August 2020 to 22:00 on the following day.

The dataset is generated from the raw data containing the GPS positions of the visitors’ smartphones detected by the station’s WiFi infrastructure. Notice that such positions are not only relative to the devices that are connected to the network, but also to those that are within the WiFi range of the system. Therefore, also the owners of smartphones that are inside the station, but are not connected to the station’s WiFi network are considered. This information is collected by several Access Points distributed over the station, all connected to the same WiFi network. The raw data contains, for each access point, the ID of the access point and the list of the connected devices (smartphones) along with their internet navigation data. Notice that these navigation data are not utilized in this work. Moreover, the Meraki system assigns a unique virtual MAC address to every connected smartphone, which is different from the physical MAC address one, to ensure the privacy of every visitor. We exploited such virtual address to identify the devices and to track them over different access points. To establish the positions of the devices in the station from the initial GPS coordinates, the latitudes and the longitudes are firstly converted into planar coordinates, and then a roto-translation into the station’s reference frame is performed. Here we assumed the upper-left corner of the map as the origin of our coordinate system. The resulting collected dataset is included in the supplementary information file *Online Resource 1*. It is represented as a comma-separated values file (.csv) organized as a table with 5 columns: the date, the time in which the visitor’s smartphone has been observed from the Meraki system, the virtual MAC address, and the coordinates in the station frame. Additional details about the process of populating and updating the heatmap will be described in Section 4.

## Case studies

In this Section, we propose four case studies illustrating the system at work in a simulated railway station. In this context, we considered the map *M* as a portion of the Roma Termini railway station (provided us by Rete Ferroviaria Italiana S.p.A.) - which is the largest and one of the most populated Italian stations - considering both randomly generated and real data about the distribution of people.

**Fig. 5:**
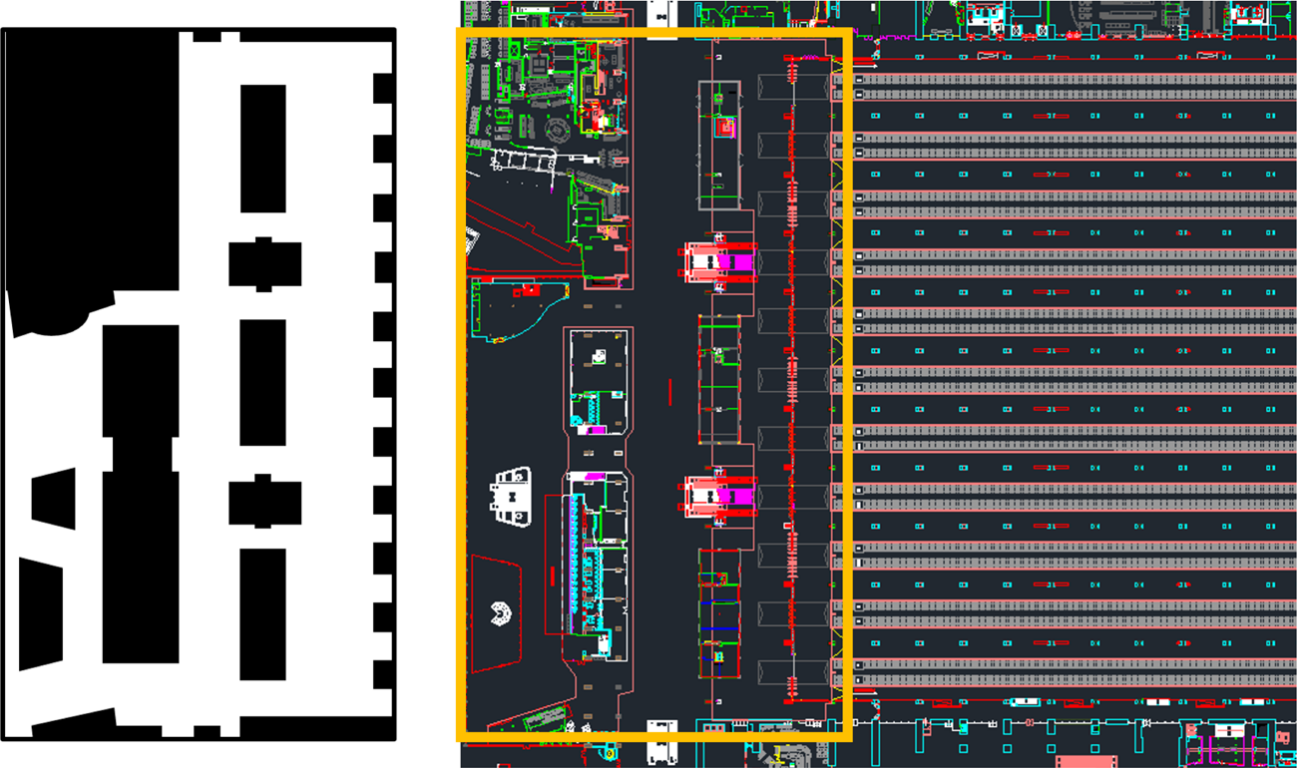
Map of the environment (left) selected from the planimetry of the Roma Termini railway station (right). The selected area (orange rectangle) represents one of the most crowded indoor sectors of the station, with an average turnout around 1000 people/per hour

A graphical representation of the environment is shown in Fig. 5. We selected a region of 100 × 172 meters (orange rectangle on the right) in front of the rails, where people usually stand waiting for the incoming trains. From that region, we also isolated shops, stairs, and walls as obstacles to be avoided by the robot during the sanitizing process (binary map on the left).

Agents can move by one pixel in any direction. Hence the set *A* includes 8 actions (4 linear and 4 diagonal) while, in case one action leads to an inconsistent location (an obstacle or out of bound), the agent stays in the current location. For the sake of clarity, a list of the main parameters is provided in Table 1.

**Table 1:** Parameters of the framework

Actor	Parameter	Value
Exp. replay	discount factor *γ*	0.99
	maximum *𝜖*	1.0
	minimum *𝜖*	0.1
	decay *𝜖*	9 ⋅ 10^− 7^
	replay buffer size	10^4^
	target network update	10^4^ steps
	main network update	4 steps
	batch size	32
WiFi server	refresh period	15 steps
Cluster of people	diameter	1 px
Robot	cleaning diameter	6 px
	speed	3 px/step
Spreading	diameter	5 px
	*μ*	0
	*σ*	0.9
Environment	dimensions	100x172 px

The framework has been implemented in Python 3.8.5, using TensorFlow 2.3.1 and Keras 2.4.3, and runs on an Intel Core i9 X-series with 3.3 GHz and 64 GB of RAM.^1^

In the first case study, we assess the system performance during the learning phase considering different numbers of robots (2 to 8 robots). In the second case, a worst-case scenario is considered, where the cleaning performance of robots are assessed considering a variable number of dynamic clusters of people randomly distributed in the station. In the third case, we propose a realistic scenario considering real data retrieved by the Roma Termini WiFi network during one day of observation. Finally, in the fourth case, we propose a comparison between our method and two additional sanitizing frameworks based on CPP [13, 46].

### Case 1: convergence and scalability

In this first case study, we designed a simulated station with randomly generated clusters of people to study the convergence and scalability of the proposed MARL approach over different team components. This training setting is intentionally designed to address a generic distribution of priorities that can be encountered by the sanitizing robots during daily cleaning activities.

In order to simulate variable and uniformly distributed clusters in a realistic environment, we set up a suitable emulator of the Roma Termini station in which each location of the map is associated with a 0.02 probability of spawning a new cluster. At the beginning of each episode, a new configuration of the map is generated so a variable number of clusters randomly spawn in different areas of the station whose positions are fixed for the entire duration of the episode. In each step of the episode, the agents acquire the current state from the central server and perform cleaning actions in order to maximize the sanitizing effect, while the server updates the map by applying the cleaning actions and by expanding the priorities through the Gaussian spreading. The episode ends when agents successfully clean up to the 98*%* of the map or when a suitable timeout is reached (400 steps in this setting). During the training process, we monitor as quantitative performance measures the overall reward, i.e., the sum of the reward earned by every agent during one episode, and the number of steps needed to accomplish the task.

**Fig. 6:**
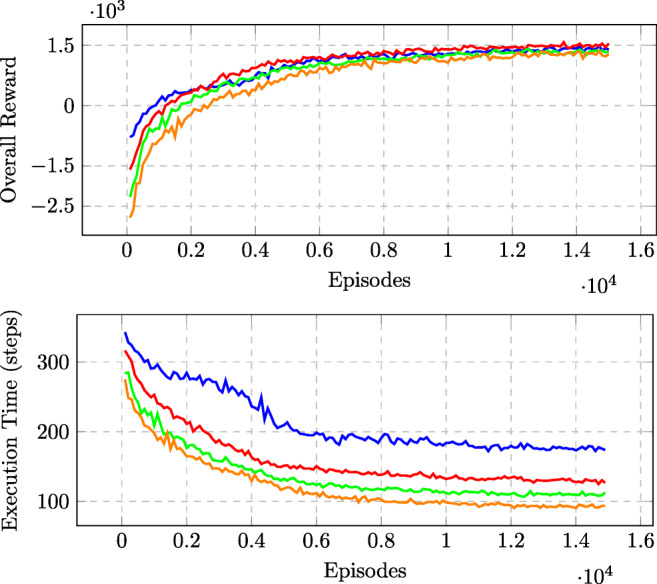
Charts of the overall reward (up) and the number of steps needed for task accomplishment (down) considering 2 (blue), 4 (red), 6 (green) 8 (orange) agents

The values recorded over 15000 training episodes are reported in Fig. 6. Considering the overall reward (Fig. 6, up), it is possible to notice that the framework performance is only partially affected by the increased number of robots: all the considered team configurations converge around a value of 1500 after almost 12000 episodes, while only the rate of convergence slightly decreases with the increased number of robots. On the other hand, the time needed to accomplish the task (Fig. 6, down) clearly decreases as the number of agents increases. Specifically, the 2 agents configuration needs 174 steps on average to accomplish the task, while the 4, 6 and 8 agents ones need 127, 112, and 94 steps, with a time reduction of 27*%*, 36*%*, and 46*%* respect to the 2 robots baseline. These two values indicate that the agents are able to cooperate during the task by minimizing the cross-agents interference (i.e., penalties due to overlapping trajectories) that becomes more likely as the number of agents increases. Specifically, since the agents are always able to successfully clean the map in the later stages of the training - and the maximum achievable reward is similar per episode (random distribution of clusters) - cooperative strategies are expected to reduce the execution time, while keeping the overall reward unchanged. On the other hand, the slight reduction in the reward indicates that the capability to cooperate is still affected by the number of agents, therefore larger teams can be associated with more frequent interference.

### Case 2: random clusters of people

**Fig. 7:**
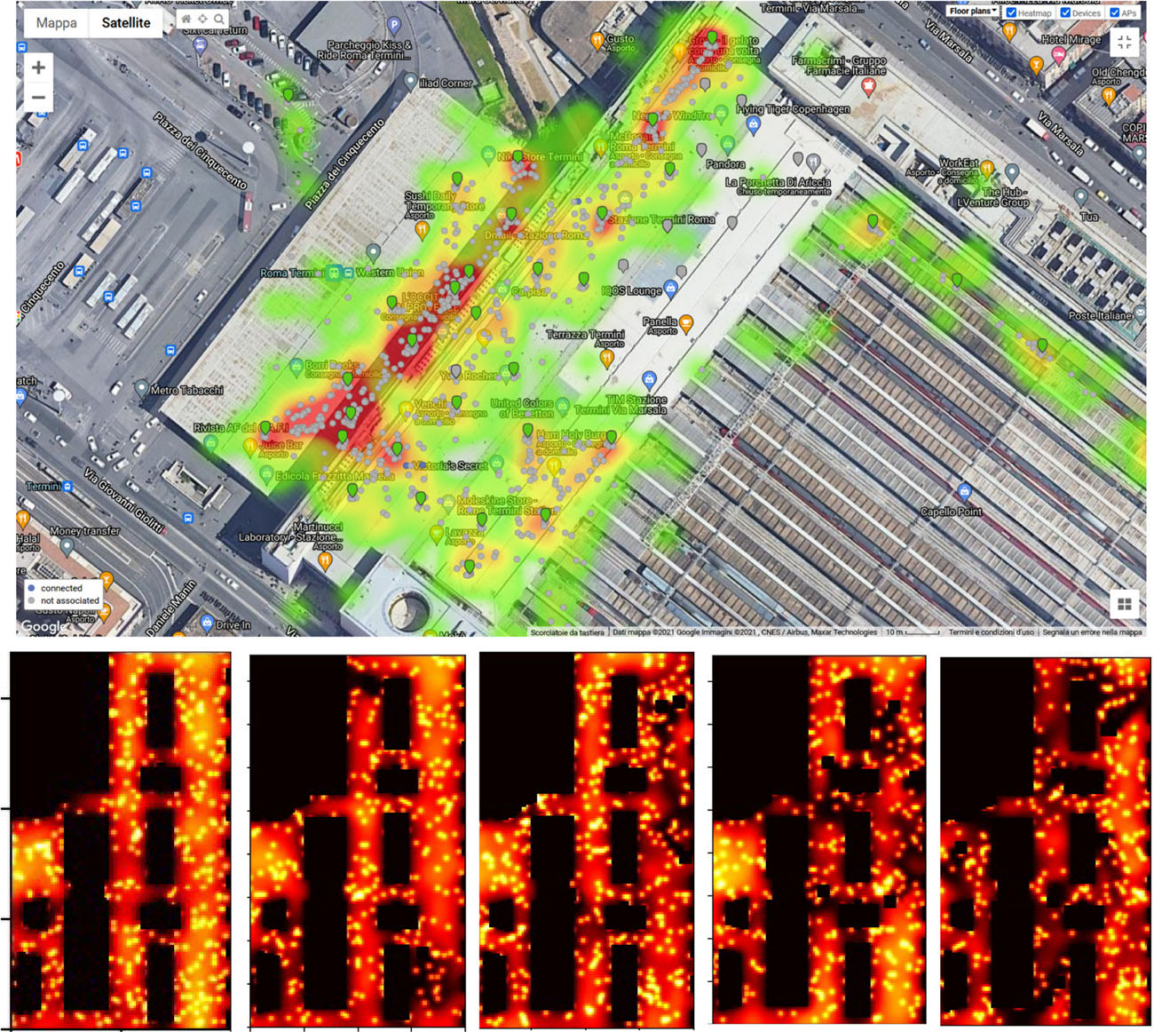
Example of the distribution of people inside the Termini station as retrieved from the Cisco Meraki WiFi network (up) and comparison of the 0 to 8 robot settings (down, from left to right) considering the simulated environment with 700 random dynamic clusters after 90 steps of execution

In order to assess the performance of the system in a realistic dynamic environment, we now propose a different case study where the trained teams are tasked to clean the railway station by considering on-line changing of priorities. In particular, we designed a simulated WiFi server that periodically updates the position of the clusters to a specific frequency while the agents have to continuously adapt their strategies in response to the changed priorities. More specifically, we designed clusters to be randomly generated on the map and randomly updated every 15 steps as to simulate the refresh of the WiFi server. Notice that the resulting uniform distribution of clusters is particularly challenging compared to a real railway station, where people are often grouped near specific areas like shops, info points, or ticket offices (see example in Fig. 7, up), hence robots can easily converge to these areas and maximize the cleaning effect. In contrast, we propose a worst-case test, where a large number of people is scattered all around the station, and robots should cover a wider area to perform the task.

In order to assess the performance of the teams, we introduce a simple metric *c*_*perc* representing the cleaning rate (in percentage) of the map as follows:

3 $$ c\_perc = ((x_{tot}-s_{curr})/x_{tot}) \cdot 100, $$

where *x*_*tot*_ = |*X*| is the number of free-obstacles cells of the map, while $s_{curr} = {\sum }_{(i,j)} s(i,j)$ is the sum of the current priorities for each cell of the heatmap. The value *c*_*perc* is then 100*%* if the map is totally clean (i.e., each cell has 0 priority) and 0*%* if every cell of the map has maximum priority. For each configuration of teams and clusters, 50 runs of 800 steps are performed. In particular, we tested 6 different environmental settings by altering the maximum number of clusters to be randomly generated in a range between 500 and 1500 clusters. This interval has been selected according to the average number of visitors per hour of the considered portion of the station (see Fig. 7, up); moreover, during the runs, the values are designed to be randomly reduced up to the 30*%* in order to simulate the people entering/exiting the station.

During this test, the server-side process defined in the previous Section (see Algorithm 2) is modified in order to simulate the WiFi refreshing period. The updated process is provided in Algorithm 3. For each run (line 2), an initialization process takes place where a set of random robots’ positions and an initial random state are created (lines 3-4). For each step of the run (line 5), when the update period is elapsed, hence new data are available from the simulated WiFi network (line 6), a set of new clusters are spawned (lines 7-8), and the updated heatmap is sent to the robots (line 9). The robots send back their updated poses once the actions are performed (line 10). Finally, after the action execution, the state is updated according to the cleaned areas and the Gaussian spreading of the priorities (11-13).

**Algorithm 3:**
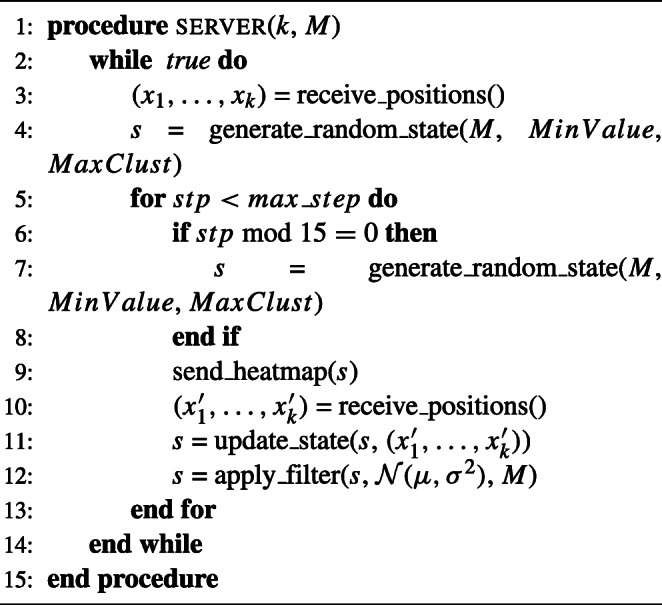
Server-side testing algorithm.

For this experiment, we have shared the video *Online Resource 2*, where a comparison of the 0 to 8 robot settings is shown considering the simulated environment with 500 random dynamic clusters.

In Table 2 we propose a comparison between the different team-settings of our method and a 0-robots baseline.

**Table 2:** Comparison between testing with different numbers of robots and passengers

Robots	Clusters	*Reward*		*c*_*perc*	
		*avg*	*astd*	*avg*	*astd*
0	500	N/A	N/A	64.09	2.54
	700	N/A	N/A	56.96	2.71
	900	N/A	N/A	51.46	2.84
	1100	N/A	N/A	47.25	2.98
	1300	N/A	N/A	43.75	3.16
	1500	N/A	N/A	40.87	3.33
2	500	11470	2392	73.63	2.60
	700	10048	3570	65.50	3.32
	900	9651	4058	59.06	3.46
	1100	8553	4602	53.73	3.74
	1300	7103	3932	48.94	3.91
	1500	4340	4113	44.49	3.89
4	500	21026	1721	79.03	2.68
	700	19422	4748	71.39	3.69
	900	19452	4239	65.63	4.04
	1100	17678	7766	59.80	4.30
	1300	16607	6380	55.08	4.79
	1500	11899	7580	49.27	4.82
6	500	26420	2166	81.85	2.64
	700	28124	2625	76.02	3.30
	900	29969	2315	71.00	3.90
	1100	31147	2306	66.62	4.44
	1300	32257	2997	62.58	4.88
	1500	32193	4654	58.44	5.24
8	500	31424	1354	84.32	2.57
	700	30713	2545	78.09	3.39
	900	29561	3367	72.23	4.17
	1100	29654	3253	67.36	4.55
	1300	29511	3679	63.11	5.10
	1500	29174	4007	59.21	5.33

In particular, for each setting, the average (avg) and the average deviation (astd) over 50 runs for both the cumulative *reward* and the cleaning percentage *c*_*perc* are collected.

These are aggregated values: since we collect the mean and the standard deviation for each one of the 50 runs, we provided *avg* as the average of the means and *astd* as the average of the deviations.

Notice, that the first 100 steps of each run are intentionally discarded, so the collected values are not affected by the transition from the initial empty environment.

The results indicate that, as expected, the cleaning performance is proportional to the number of robots and inversely proportional to the number of people in the station. On average, the value of *c*_*perc* reduces by 5.3*%* for every 200 additional clusters and increases by 6.8*%* between the baseline and the 2-robots setting and by an additional 4.4*%* for every 2 robots added. Analogously, also the collected *reward* increases according to the size of the team. This suggests that trained robots are able to effectively cooperate during the execution of the sanitization task. This improved performance can also be noted in Fig. 7 (down), where the evolution of the heatmap for different configurations of the teams is compared to the 0-robots baseline (first from the left). In this respect, it is possible to notice that, starting from the 2 robot configuration (second from the left), the number of yellow spots (i.e., high-priority areas) significantly reduces, while the cleanliness of the map increases with the number of robots (from left to right).

### Case 3: dynamic clusters from real data

Thanks to the collaboration with the Italian railway infrastructure manager Rete Ferroviaria Italiana, we received one day of real data recording collected the 13 August 2020 by the Roma Termini Cisco Meraki WiFi infrastructure. In particular, the collected data includes the GPS positions of the smartphones owned by the visitors along with an associated id that allows devices to be tracked along the station with around 3 meters of accuracy.

In this case study, the aim is to perform extensive tests of our solution using the received real-data recording showing the behavior of the system in relation to different team settings (from 2 up to 8 robots) and different distributions of people from the different time periods within one day of execution.

In particular, although the station is 24h open, the experiments have been carried out considering a range of time between 6:00 and 22:00 hours. This time interval is associated with a relevant density of people because of the open services and shops, and because of the greater traffic of trains due to tourism, leisure and the work-related commuting in Rome [47]. At the beginning of the experiment, to prevent the dependence of the test from the robot starting point, the initial positions of the agents are randomly chosen between the free-obstacle spaces of the station, while for the environment, we consider a blank initial condition, in which the station is completely clean.

Analogously to the previous case (Section 4.2), also in this case study, we maintain a 1:1 relation between minutes and steps. We assume a 15 minutes (15 steps) refresh time for the WiFi server to update the heatmap information with the new positions of people from the recorded data. In particular, here we consider a server-side implementation similar to the one proposed in Algorithm 3, but we exploit the real recorded data instead of the randomly generated ones (*generate*_*random*_*state* function in lines 4 and 7). If multiple positions are associated with the same id within the 15 minutes interval, the WiFi network is able to collect different waypoints from a visitor’s motion, then multiple hot-points are added to the heatmap in order to drive the robots toward the sampled trajectory. A video for this experiment is also available as *Online Resource 3*, showing a comparison between the 0 to 8 robot settings.

**Fig. 8:**
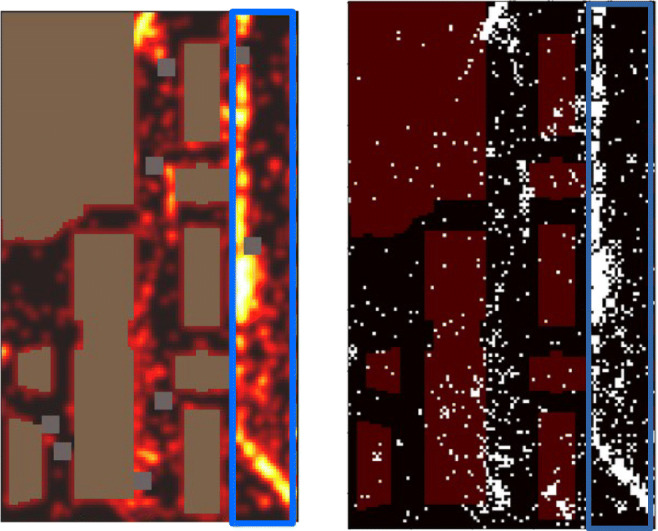
Example of our heatmap with a team of eight sanitizing robots (left) and the distribution of people in the Termini station, obtained from GPS position data present in Cisco Meraki WiFi Database (right). The blue rectangle illustrates the zone of the station between the railway platforms and the gates, where there is a high probability of finding a gathering of people

Since, in this case, the distribution of people is not uniform, in addition to the whole map performance, we also monitor the cleaning effect on the most visited area of the station. Specifically, we selected the most frequented area *Z* as the portion of the map that lays between the platforms of the trains and their associated access gates (blue rectangle in Fig. 8).

By empirically analyzing the recorded data, we have verified that in *Z*, there is far greater probability of finding a gathering of passengers during the day (white pixels in Fig. 8, right). This condition mainly depends on the presence of the new checkpoints installed near the gates during the period of the Covid-19 pandemic, which people have to pass in order to access the platforms and wait for the trains.

In order to assess the performance on *Z*, we defined a specific *c*_*perc*_*z*_ value similarly to the one proposed in (3). In this case, we set *x*_*tot*_ = |*Z*| as the number of free-obstacle cells of the map in the zone *Z*, while $s_{curr} = {\sum }_{(i,j)\in Z} s(i,j)$ is the sum of the current priorities for each cell of the heatmap in *Z*.

**Fig. 9:**
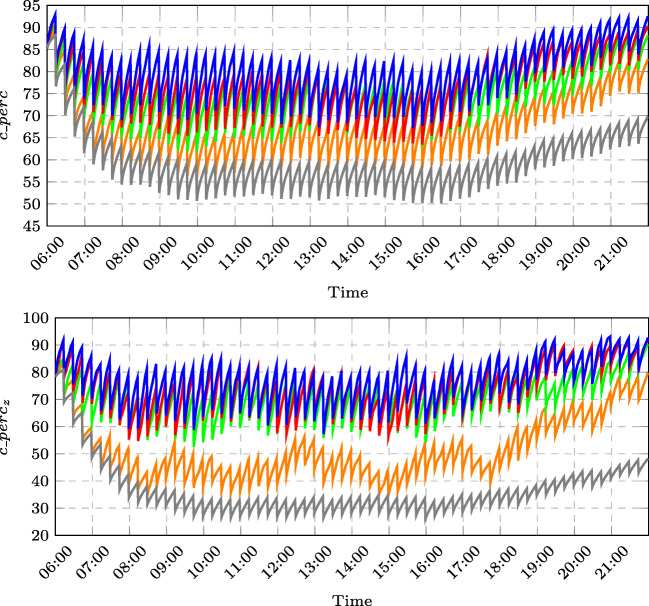
Charts of *c*_*perc* (up) and *c*_*perc*_*z*_ (down), considering 2 (orange), 4 (green) 6 (red), 8 (blue) agents and without any robot (gray)

Our experiment is performed as follows: firstly, we designed a baseline setup in which the real recorded data are simulated without robots, hence no cleaning is considered. Then this 0-robot baseline is compared with respect to the different teams. In Fig. 9, the performance in terms of *c*_*perc* (up) and *c*_*perc*_*z*_ (down) for teams of 2 (orange), 4 (green), 6 (red) and 8 (blue) agents are compared with respect to the 0 robots baseline (gray). It is possible to notice that, as expected, the cleaning performance for the whole map (Fig. 9, up) improves rapidly from the baseline up to the 2 and 4 robots settings, becoming less evident from the 4 to the 8 robots settings. This behavior is more evident in *Z* (Fig. 9, down) where the 4 to 8 robots settings strongly outperform the baseline and the 2 robots team. This increment can also be noted from Table 3, where the average *c*_*perc* and *c*_*perc*_*z*_ for the different teams over the whole day of execution are shown. By considering the whole map, there is a 20.36*%* increment of *c*_*perc* between the baseline and the 8 robots setting. This increment is almost doubled for the *Z* area where *c*_*perc*_*z*_ increases up to 40.28*%* between the baseline and the proposed framework.

**Table 3:** Percentage of the cleaning in the real-case testing

Team	avg *c*_*perc*	avg *c*_*perc*_*z*_
0	59.74	36.71
2	67,81	51,63
4	74.74	70.43
6	77.01	74.04
8	80.10	76.99

**Fig. 10:**
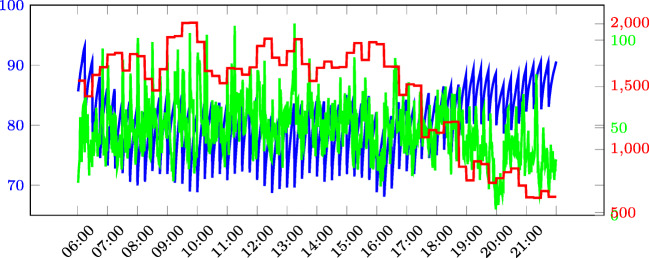
Charts of the overall reward (green) and the number of passengers/visitors of the station (red) the percentage of cleaning *c*_*perc* for a team of 8 robots in an interval of time between 6.00 and 22.00 o’clock (blue)

In Fig. 10 we show details about the 8 robots setting, where the relation between the overall reward (green), the cleaning percentage *c*_*perc* (blue), and the number of visitors in the station (red) is emphasized. It is noticeable that the three values fluctuate with a frequency of 15 steps (equivalent to 15 minutes). This behavior is particularly evident in the *c*_*perc* value, whose sawtooth-like behavior directly depends on the 15 minutes refreshing time of the server-side process that continuously updates the heatmap adding new hot points according to the recorded data. In the interval 6:00-18:00, which is associated to an increased number of people along with a wider distribution of priorities, the overall reward increases while the value of *c*_*perc* decreases down to a minimum value of 70*%*. Those behaviors are expected because of the increased complexity of the sanitizing problem associated to the rush hours in the station. In contrast, after 18:00 o’clock, when the number of people in the station significantly decreases, the value of *c*_*perc* increases again up to a maximum value of almost 93%.

In order to further highlight the relation between the cleaning performance and the time slots during a single day of execution, we designed an additional experiment where the recorded data are divided into intervals of 2 hours. In particular, we deploy a team of 8 robots in 8 partitions of time from 6:00-8:00 to 20:00-22:00. In this case, we applied for each time interval the server-side Algorithm 3, where the update of the heatmap is executed with real recorded data instead of the randomly generated ones (*generate*_*random*_*state* function in line 4 and 7), as described above.

Notice that, in order to decouple each time interval from the previous ones, every simulated interval is started by considering a blank initial condition, in which the station is completely clean. This process allows us to analyze the workload of the team over the different intervals, which is independent from the performance in the previous slots.

**Fig. 11:**
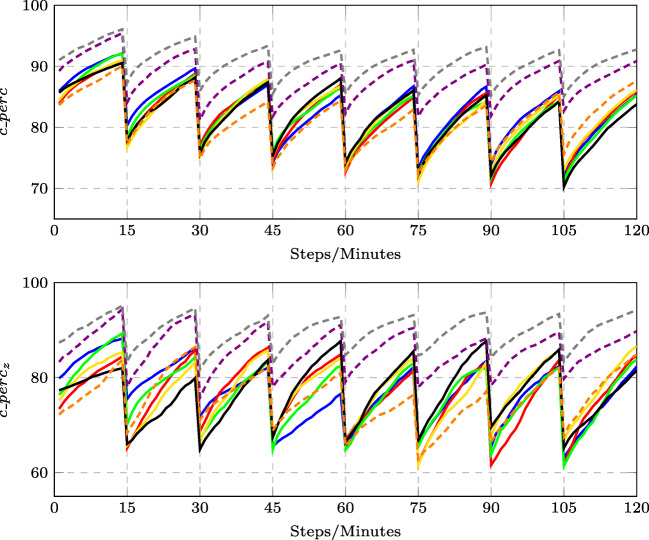
Charts of the value of *c*_*perc* (up) and *c*_*perc*_*z*_ (down), achieved by a team of 8 robots in different time intervals: range 6:00-8:00 (blue), 8:00-10:00 (red), 10:00-12:00 (green), 12:00-14:00 (yellow), 14:00-16:00 (black), 16:00-18:00 (orange), 18:00-20:00 (dashed violet), 20:00-22:00 (dashed gray)

In Table 4, we provide for each interval the average (avg) and the average deviation (astd) for both the cumulative reward and the cleaning percentages *c*_*perc* and *c*_*perc*_*z*_ over 50 runs. Here it is possible to notice that the performance strongly depends on the number and the distribution of people in the station (workload). Specifically, the 8 robots team shows the worst performance in the three ranges of time (8:00-10.00, 12:00-14:00, and 16:00-18:00), which are associated to the higher quantity of people. In these cases, the average value of *c*_*perc* is always lower than 82*%*. In contrast, better performance are associated to less populated intervals (18:00-20:00 and 20:00-22:00) with a average *c*_*perc* over 87*%*. This behavior is also shown in Fig. 11 where a single run of the framework is provided. In order to ensure the same performance of cleaning in every interval of time, a possible solution can be to provide a cleaning strategy where the number of robots on the team can be dynamically adapted depending on the specific intervals.

**Table 4:** Results of a team composed of 8 robots in different time intervals

*rng*	*Reward*		*c*_*perc*		*c*_*perc*_*z*_	
	*avg*	*astd*	*avg*	*astd*	*avg*	*astd*
6-8	5917	342.55	82.57	4.73	77.90	6.21
8-10	6353	353.92	81.86	4.96	76.48	6.47
10-12	6120	648.00	82.27	4.29	76.19	6.14
12-14	6460	266.21	81.91	4.57	76.76	6.00
14-16	5692	571.00	81.81	4.97	76.48	6.28
16-18	5289	586.36	82.61	3.77	78.05	4.85
18-20	5083	294.38	87.99	3.00	84.79	4.28
20-22	4207	205.06	90.20	2.57	87.85	3.39

### Case 4: comparison with alternative techniques

In this section, we propose a comparison between our MARL framework and two typical CPP-based methods for robot cleaning. The first method is a spiral-based [46] exploration of the environment, which is divided into *k* non-overlapping sub-areas of equal dimensions, each one assigned to a single robot. Sub-areas are partitioned by rectangular decomposition and such partitions are covered by spiral paths. The second method is boustrophedon-based [13] exploiting a modified version of the boustrophedon environment decomposition for partitioning and boustrophedon paths for coverage.

**Fig. 12:**
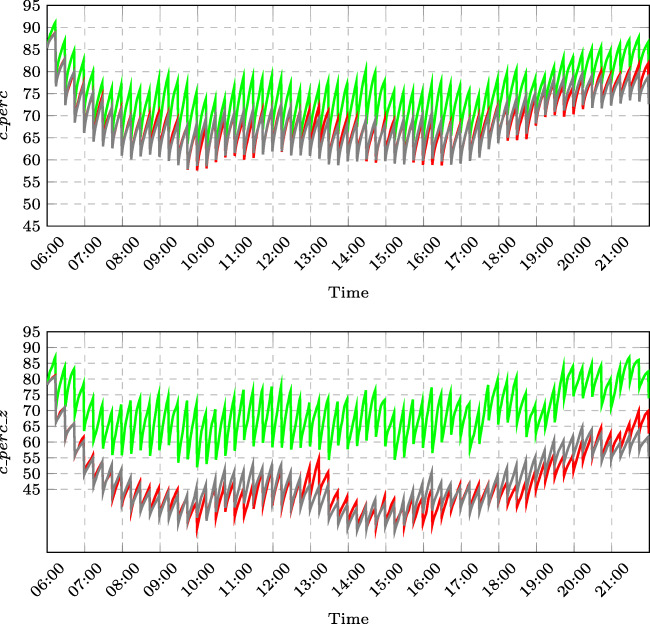
Comparison between the proposed MARL framework (green) and the alternative spiral-based (red) and boustrophedon-based (gray) CPP ones considering both *c*_*perc* (up) and *c*_*perc*_*z*_ (down) values. In all settings a team of 4 robots is deployed

The comparison has been carried out considering the real-data from the previous case study and teams of 4 robots for each method. Also in this case the values of *c*_*perc* and *c*_*perc*_*z*_ are used to assess the cleaning performance. The results of the tests are shown in Fig. 12. The proposed MARL framework (green) outperforms the 2 alternatives in the whole station (+ 5*%* in average) as well as in the *Z* area (+ 22*%* in average). This increment is particularly relevant in *Z*, where cleaning performance is always greater than 50*%* while both the spiral-based (red) and the boustrophedon-based (gray) performance are often under 45*%* of cleaning. This difference is mainly due to the flexibility of the MARL framework, which is able to adapt the cleaning strategy of the robots depending on the environment, while the CPP-based methods are mainly based on pre-defined coverage strategies.

## Conclusions

In this work, we proposed a scalable Deep Q-Learning approach to multi-robot sanitization of railway stations. The proposed framework exploits real-time data about the distribution of people in the environment to generate a priority map of the areas to be sanitized. Such map is then exploited by a set of robots, each endowed with a convolutional neural network, to learn an effective high-level strategy which optimizes the overall sanitization processes. We tested the proposed framework in a realistic simulated scenario, which was designed in cooperation with the Italian railway infrastructure manager (RFI S.p.A.), considering the largest and most populated Italian railway station (Roma Termini) as a case study. We assessed the performance of the proposed framework in different case studies, firstly by considering a worst-case scenario where random clusters of people are scattered along the station, then by considering a more realistic setting in which the distribution of people is retrieved from a one-day data recording provided by the Meraki Cisco System WiFi Network of Roma Termini. The collected results show that the proposed framework is capable of generating long-term strategies for a team of robots in order to suitably sanitize large indoor environments, such as railway stations. We also discussed the scalability of the proposed method with respect to the number of involved robots and the density of people in the station. Finally, we illustrated the effectiveness of the approach with respect alternative CPP-based methods for robot cleaning. As a limitation of the approach, we can observe that the time needed to train multiple DQNs for our MARL framework increases with the number of robot (each new robot is associated with a new DQN). Notwithstanding the offline and distributed training process, such increment may impair the deployment of the framework in very large robotic teams. To mitigate this problem, the networks can be distributed on different machines - our DTDE approach is particularly suited for such solution - and epsilon-greedy mechanisms [48] may be deployed for action selection instead of the current uniform sampling. In this paper, we focused on the generation of cleaning policies assuming that the robots were always able to reach and completely clean target areas. On the other hand, navigation and cleaning failures (e.g., due to the presence of people or unexpected obstacles) should also be addressed in real-world implementation. As a future work, we plan to deploy and assess the proposed approach in more complex scenarios where also cleaning failures and social navigation issues are considered. To this end, routing algorithms [49, 50] and human action recognition methods [51–54] will be investigated and integrated. We are also interested in learning methods to adapt the dimension and the strategies of the robot team with respect to the heatmap status in order to reduce power consumption and maintenance costs, while preserving the quality of the sanitization.

## Footnotes

1 The source code of the system is available at the following link: https://github.com/Tavano1/MultiRobot_Sanitization_Railway
